# Noninvasive versus invasive mechanical ventilation for immunocompromised patients with acute respiratory failure: a systematic review and meta-analysis

**DOI:** 10.1186/s12890-016-0289-y

**Published:** 2016-08-27

**Authors:** Tao Wang, Lixi Zhang, Kai Luo, Jianqiang He, Yong Ma, Zongru Li, Na Zhao, Qun Xu, Yi Li, Xuezhong Yu

**Affiliations:** 1 Emergency Department, Peking Union Medical College Hospital, Beijing, 100730 China; 2 Department of Cardiology, Peking Union Medical College Hospital, Beijing, 100730 China; 3 Department of Epidemiology and Biostatistics, Institute of Basic Medical Sciences Chinese Academy of Medical Sciences, School of Basic Medicine Peking Union Medical College, Beijing, 100005 China; 4 Department of Pneumology, Peking Union Medical College Hospital, Beijing, 100730 China; 5 Department of Anesthesiology, Beijing Obstetrics and Gynecology Hospital, Capital Medical University, Beijing, 100026 China

**Keywords:** Noninvasive mechanical ventilation, Invasive mechanical ventilation, Acute respiratory failure, Immunocompromised patients, Systematic review, Meta-analysis

## Abstract

**Background:**

To determine the effects of noninvasive mechanical ventilation (NIV) compared with invasive mechanical ventilation (IMV) as the initial mechanical ventilation on clinical outcomes when used for treatment of acute respiratory failure (ARF) in immunocompromised patients.

**Methods:**

We searched PubMed, EMBASE, the Cochrane Central Register of Controlled Trials (CENTRAL), the Chinese Biomedical Literature Database (CBM) and other databases. Subgroup analyses by disease severity and causes of immunodeficiency were also conducted.

**Results:**

Thirteen observational studies with a total of 2552 patients were included. Compared to IMV, NIV was shown to significantly reduce in-hospital mortality (OR 0.43, 95 % CI 0.23 to 0.80, *P* value = 0.007) and 30-day mortality (OR 0.34, 95 % CI 0.20 to 0.61, *P* value < 0.0001) in overall analysis. Subgroup analysis showed NIV had great advantage over IMV for less severe, AIDS, BMT and hematological malignancies patients in reducing mortality and duration of ICU stay.

**Conclusions:**

The overall evidence we obtained shows NIV does more benefits or at least no harm to ARF patients with certain causes of immunodeficiency or who are less severe.

**Electronic supplementary material:**

The online version of this article (doi:10.1186/s12890-016-0289-y) contains supplementary material, which is available to authorized users.

## Background

The immunocompromised condition is defined as a state of subnormal immune response of the host to a foreign antigen, which could be congenital (primary) or acquired (secondary) [1], with primary immunodeficiencies caused by gene mutations and secondary caused by malignancy, chemotherapies of malignancy, malnutrition, aging, viral infection, immunosuppressive medication for treatment of a variety of disorders such as autoimmune disease and organ transplantation. The number of immunocompromised patients has increased dramatically over recent decades [2]. In spite of better antimicrobial agents and preventive measures, infections continue to be one of the most frequent complications in immunocompromised patients and have a high mortality rate of 30 to 90 % [2], with the highest when acute respiratory failure (ARF) occurs. Thus, early diagnosis and proper intervention are essential for better outcomes. Noninvasive mechanical ventilation (NIV) and invasive mechanical ventilation (IMV) are two approaches for providing supplemental oxygen for patients with relatively severe ARF. NIV has gained more and more popularity since its first application in 1980s [3], and is now widely accepted as a first-line intervention for certain forms of ARF, including acute exacerbation of chronic obstructive pulmonary disease (AECOPD) and cardiogenic pulmonary edema [4–14].

Although the use of NIV as a first-line strategy for immunocompromised patients with acute respiratory failure was recommended by Canadian Critical Care Society Noninvasive Ventilation Guidelines Group(Grade 2B: weak recommendation and moderate evidence quality) [11], recent studies showed noninvasive ventilation might not be the appropriate choice for all immunocompromised patients [15–17]. The choice of NIV versus IMV for immunocompromised patients, especially for relatively severe ARF, is still under debate. On the other hand, there are studies indicating that the use of NIV as the initial treatment for certain population may delay intubation, which may increase mortality and cost of health care [17–20]. Identifying the proper candidates and evidence for the effect of NIV is of great importance for better outcome of immunocompromised patients with ARF. This review will provide a systematic review of the evidences to determine the effects of NIV compared to IMV on clinical outcomes when used for treatment of acute respiratory failure in immunocompromised patients.

## Methods

### Search strategy

We searched the following databases (Additional file 1): PubMed, EMBASE, the Cochrane Central Register of Controlled Trials (CENTRAL), and Chinese Biomedical Literature Database (CBM, in Chinese). We also searched other resources including Web of Science (WOS), National technical information service conference proceedings (NTIS), Open Grey (OG), and conference proceedings for relevant abstracts, online clinical trial registers for ongoing and recently completed studies including Controlled Clinical Trials (http://www.controlled-trials.com/), government registries (http://www.clinicaltrials.gov), and World Health Organization registries (http://www.who.int/trialsearch/). There was no study type, language, date, or publication type restrictions. We searched the bibliography of all included studies and requested original data from the primary authors when necessary. The most recent search was conducted on April 15th, 2016.

### Inclusion criteria

We included randomized controlled trials (RCTs) and non-RCTs. We accepted the definition of acute respiratory failure as the state when ratio of the partial pressure of arterial oxygen to the fraction of inspired oxygen (PaO2/FiO2) ≤ 300 mmHg. For studies enrolling patients with other causes for mechanical ventilation, we stipulated that a minimum of 85 % of patients must have ARF to meet the inclusion criteria. Alternatively, patients were considered as immunocompromised when clinically diagnosed as: 1) HIV-infected individuals (with or without acquired immune deficiency syndrome, AIDS), 2) individuals on immunosuppressive therapy (e.g., cytotoxic agents, glucocorticoids, etc.), 3) transplantation individuals (i.e., solid organ transplantation or bone marrow transplantation), 4) hematologic cancers, 5) certain trauma or surgery (splenectomy), 6) secondary to metabolic diseases (e.g., malnutrition, noncontrolled diabetes, uremia). The intervention group included patients who received NIV as the initial mechanical ventilation technique, in addition to standard medical care, irrespective of whether IMV was also used later during the hospital stay. The control group included patients who received IMV as the initial MV technique. Patients with absolute contraindications of NIV including respiratory arrest and inability to fit the mask, or with underlying pathologies where NIV and IMV is both contraindicated, such as facial trauma, were excluded. We included studies in which at least one of the review-defined outcomes was identified. The primary outcomes were all-cause mortality, including mortality in hospital or intensive care unit (ICU), and 30-day mortality after ICU admission. The secondary outcomes included duration of hospitalization and ICU stay, nosocomial infections, and duration of mechanical ventilation.

### Data extraction and study quality

Two authors (JH and YM) independently assessed studies for inclusion retrieved from electronic searches and other resources and extracted data from the included studies. In cases of ambiguity or insufficient data, we requested additional information from study authors. Disagreements were ultimately resolved by a third author (YL). We used a standardised data extraction form to collect the following data: 1) General information: study ID, title, authors, source, language and year of publication, country, and source of funding. 2) Study characteristics: study type, hospital settings, and dates of study. 3) Participants: age, sex, diagnosis standard and cause of ARF and the immune status, sample size, baseline physiological variables including Acute Physiology and Chronic Health Evaluation (APACHE II) scores, Simplified Acute Physiology Scores (SAPS II) [21] and details of respiratory state on admission. 4) Interventions: intervention used in each group and number of each group; indications, contraindications, settings and duration of intervention. 5) Outcomes: mortality (in ICU, in hospital and 30-day), duration of mechanical ventilation, ICU stay and hospitalization, number of participants with development of major complications or nosocomial infections, numbers experiencing each outcome, and information of follow-up. 6) Methodological quality: items in the Cochrane Collaboration’s tool [22] and Newcastle Ottawa Scale for assessing risk of bias (Additional file 1) [23].

Two review authors (TW, LXZ) independently assessed the risks of bias in included studies. We used the adapted Newcastle Ottawa Scale to assess the risks of bias, including selection, comparability and outcome/exposure (Additional file 1) [23]. We also explored other risks of bias such as reporting bias. We then analyzed the quality of evidence following the Grading of Recommendations Assessment, Development and Evaluation (GRADE) methodology [24, 25].

### Statistical analysis

We used random-effect models throughout the analysis to take account of the between-study variance in our findings. All analyses were performed on R3.2 with the meta and metafor packages. We considered *P* values < 0.05 to be statistically significant. When outcomes were dichotomous, we used unadjusted odds ratio (OR) for non-RCTs (cohort and case-control studies); when outcomes were continuous, we used mean difference (MD); each was provided with 95 % confidence intervals (95 % CIs).

We followed the recommendations of the Cochrane Handbook for Systematic Reviews of Interventions [22] in assessing the impact of heterogeneity. In general, we interpreted an I^2^ value > 60 % as having substantial heterogeneity and meta analyses cannot be done; an I^2^ value ≤ 40 % would suggest little concern about statistical heterogeneity. If data was sufficient, we would undertake the following subgroup analyses for each comparison:

1. Severity of disease: based on SAPS II [21] at admission. Studies were classified into less severe group when a mean of SAPS II < 60 and more severe group when a mean of SAPS II ≥ 60. Participants in more severe group were considered to have more severe disease than those in less severe group. In addition, disease severity could also be assessed by Sequential Organ Failure Assessment (SOFA) [26] and PaO2/FiO2.
2. Causes of the immunocompromised status.

## Results

**Fig. 1:**
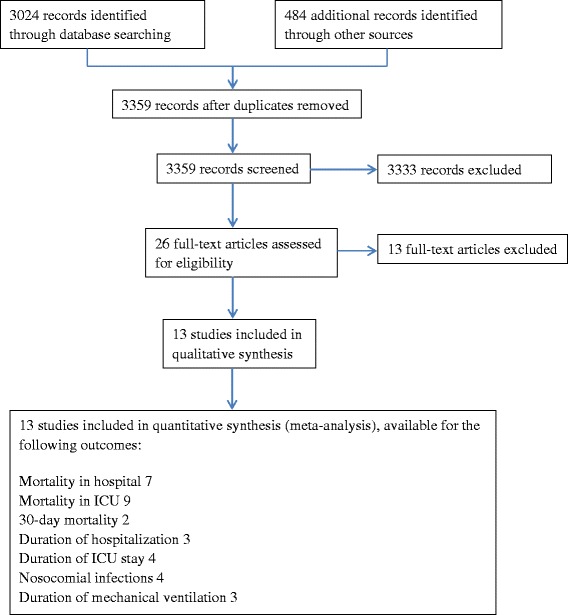
Study flow diagram

We retrieved 3359 records from the electronic database searches (Fig. 1 for a flow diagram of studies identified). Thirteen non-RCTs [17, 20, 27–37] met all of the inclusion criteria with a detailed description of each available in Table 1. There were one prospective cohort study [20], three retrospective cohort studies [28, 31, 34] and nine retrospective case-control studies [17, 27, 29, 30, 32, 33, 35–37]. A total of 2552 patients were included in our final analysis. Sample size in each study ranged between 15 and 1302. One study included children with a mean age of 9 years old [33]. The rest recruited adults with age ranged from 17 to 82. All studies met the diagnosis criteria for acute respiratory failure and immunocompromised status. Main types of ARF included adult respiratory distress syndrome/acute lung injury (ARDS/ALI), infectious pneumonia, chronic obstructive pulmonary disease (COPD), and pulmonary edema. Causes of patients’ immunocompromised status included AIDS, hematologic malignancies, solid tumors, bone marrow transplantation (BMT), chemotherapy and receipts of immunosuppressive medications. All included studies compared NIV with IMV as the first mechanical ventilation technique in immunocompromised patients with ARF. Settings in the NIV group were as follows: (1) Ventilation modes: two studies [27, 32] used continuous positive airway pressure (CPAP) ventilation only; three [33, 34, 36] solely used bi­level positive airway pressure (Bi­PAP); another five [17, 20, 28, 30, 37] solely used pressure support ventilation (PSV); another one [31] used both CPAP and Bi-PAP; no description was given in the remaining studies. (2) Interfaces: full face mask, face mask, nasal mask or helmet. (3) Duration and continuity: eight studies applied NIV continuously for the first 24 h in the NIV group. Four studies didn’t report the duration of mechanical ventilation. (4) Positive End Expiratory Pressure (PEEP): in most studies, PEEP ranged between 3–10 cmH_2_O, adjusted with ventilation flow, pressure support and other settings to achieve a proper clinical outcome, including a pulse oximetry oxyhemoglobin saturation ≥ 95 %, an oxygen saturation ≥ 90 %, FiO2 ≤ 0.6, an exhaled tidal volume of 8 to 10 ml/ kg or a respiratory rate ≤ 25 breaths/min. A portion of patients who initially received NIV switched to IMV due to NIV failure, with a mean intubation rate of 49.5 %, ranging from 25.8 to 78.3 %. For the IMV group, however, only three studies gave a description for ventilation settings, two of which used volume-controlled ventilation, the other used pressure controlled ventilation. Supportive treatments were described in three studies, including use of antimicrobial agents, diuretics, bronchodilators, granulocyte-colony stimulating factor, dialysis, and parenteral nutrition, etc.

**Table 1:** Characteristics of the included studies

Study	Study design	Settings	Sample size (NIV/IMV)	Cause of immunodeficiency	SAPS II (SD orrange)	Number of NIV patients that switched to IMV (%)	Outcomes
Azoulay 2001	Cohort, single-centered	France, ICU	96 (48/48)	Hematologic malignancy or solid tumors	NIV: 47 (38–60)	Not specified	1. 30-day mortality
					IMV: 44.5 (36–59)		2. Nosocomial infections
Azoulay 2003	Case-control, single-centered	France, ICU	15 (7/8)	Hematological malignancy	NS	4 (57.1)	1. Mortality (in ICU)
Azoulay 2004	Case-control, single-centered	France, ICU	148 (79/69)	Hematological malignancy, allogeneic BMT, solid tumors, chemotherapy	NS	45 (57.0)	1. Mortality (in hospital)
B-M 2013	Case-control, single-centered	Spain, ICU	41 (35/6)	Hematological malignancy	63 (18)	14 (40.0)	1. Mortality (in hospital)
							2. Mortality (in ICU)
							3. Duration of ICU stay
							4. Duration of hospitalization
							5. Nosocomial infections
							6. Duration of mechanical ventilation
Confalonieri 2002	Cohort, single-centered	Italy, ICU	48 (24/24)	AIDS	NIV: 37 (9)	8 (33.0)	1. Mortality (in ICU)
					IMV: 38 (5)		2. Duration of ICU stay
							3. Duration of hospitalization
							4. Nosocomial infections
							5. Duration of mechanical ventilation
Depuydt 2004	Cohort, single-centered	Belgium, ICU	78 (26/52)	Hematological malignancy and allogeneic BMT	NIV: 46	18 (69.2)	1. Mortality (in hospital)
					IMV: 46		
Depuydt 2010	Cohort, single-centered	Belgium, ICU and general medical units	91 (24/67)	Hematological malignancy and allogeneic BMT	NIV: 52 (15)	18 (75.0)	1. Mortality (in ICU)
					IMV: 65 (18)		2. Mortality (in hospital)
							3. Duration of ICU stay
Gachot 1992	Case-control, single-centered	France, ICU	45 (36/9)	AIDS	NS	11 (30.6)	1. Mortality (in ICU)
							2. Mortality (in hospital)
Gristina 2011	Case-control, multicenter	Italy, ICU	1302 (274/1028)	Hematologic malignancy	NIV: 49 (16)	126 (46.0)	1. Mortality (in ICU)
					IMV: 58 (18)		2. Mortality (in hospital)
							3. Duration of ICU stay
							4. Duration of hospitalization
							5. Duration of mechanical ventilation
							6. Nosocomial infections
Molina 2012	Case-control, multicenter	Spain, ICU	300 (131/169)	Hematological malignancy and BMT	NS	79 (60.3)	1. Mortality (in ICU)
Pancera 2008	Case-control, single-centered	Italy, PICU	239 (120/119)	Hematologic malignancy or solid tumors	NS	31 (25.8)	1. Mortality (in ICU)
							2. 30-day mortality
Rabitsch 2005	Case-control, single-centered	Austria, ICU	82 (35/47)	Autologous or allogeneic BMT for hematological malignancies	NIV: 62 (49–84)	24 (68.6)	1. Mortality (in hospital)
					IMV: 68 (51–87)		
Turkoglu 2013	Case-control, single-centered	Turkey, ICU	67 (46/21)	Hematological malignancies	NS	36 (78.3)	1. Mortality (in ICU)

*Abbreviations*: *AIDS* acquired immune deficiency syndrome; *BMT* bone marrow transplantation; *ICU* Intensive Care Unit; *PICU* Pediatric Intensive Care Unit; *USA* United States of America; *NIV* Noninvasive mechanical ventilation; *IMV* Invasive mechanical ventilation; *SAPS II* Simplified Acute Physiologic Score II; *NS* Not stated; *SD* Standard deviation

Five studies were excluded for the fact that there was no or improper invasive mechanical ventilation group set as control group among these studies [38–42]. Another common reason was that invasive mechanical ventilation was not studied as a comparison but rather an outcome of the non-invasive ventilation [43, 44]. Two more studies were excluded since attempts to obtain original data from the author concerning subgroup data were unsuccessful [45, 46]. Two studies were excluded since less than 85 % of participants who were diagnosed as ARF on admission [47, 48]. One was excluded because participants were not restricted within patients with ARF [49]. In addition, baseline of PaO2/FiO2) was higher than 300, which has already exceeded the upper limit of current definition of ARF. One was excluded since it used a different definition of 30-day mortality [50].

### Study quality

We used the adapted Newcastle Ottawa Scale to assess the risk bias in cohort/case-control studies in our review, as described in the Methods section. The four cohort studies scored between 7 and 8 (out of a 9 points), with one study at low risk of bias and three at medium risk. The other nine case-control studies scored between 3 and 6 (out of 8 points), all assessed as high risk of bias but one as medium (see Additional file 1).

We defined main primary outcomes to assess the quality of evidence using the GRADE methodology (Table 2). The main factor that may downgrade the levels of quality was the non-RCT study design in all included studies, which share the inherent limitations of the design and implementation compared to RCTs. Another factor was the inconsistency of results across the small number of included trials. The substantial heterogeneity may be attributed to methodological variations among studies and clinical variations among participants. Although the definition of ARF fulfilled the inclusion criteria, substantial heterogeneity existed in the types of ARF. The paucity of studies made us unable to conduct subgroup analyses or meta regression analyses to explore causes of such heterogeneity. Few studies reported estimates of effect after adjustment by multiple variables. In addition, some included studies had small sample sizes, which made them less representative of the exposed population. There were also factors that upgraded the levels of quality for the outcome (30-day mortality) due to relatively large outcome events (Table 2).

**Table 2:** Summary of main findings

Patient or population: Immunocompromised patients with acute respiratory failure Setting: ICUs, General medical units. Intervention: Noninvasive mechanical ventilation Comparison: Invasive mechanical ventilation						
Outcomes	Anticipated absolute effects^a^ (95 % CI)		Relative effect (95 % CI)	No. of participants (studies)	Quality of the evidence (GRADE)	Comments
	Risk with invasive mechanical ventilation	Risk with Noninvasive mechanical ventilation				
Mortality in hospital	624 per 1000	416 per 1000 (276 to 570)	OR 0.43 (0.23 to 0.80)	1787 (7 observational studies)	⨁◯◯◯ VERY LOW^b^	
Mortality in hospital- Less severe subgroup	584 per 1000	496 per 1000 (431 to 558)	OR 0.70 (0.54 to 0.90)	1380 (2 observational studies)	⨁⨁◯◯ LOW	
Mortality in ICU	549 per 1000	339 per 1000 (226 to 464)	OR 0.42 (0.24 to 0.71)	2148 (9 observational studies)	⨁⨁◯◯ LOW^b,c^	
Mortality in ICU- AIDS subgroup	576 per 1000	230 per 1000 (98 to 440)	OR 0.22 (0.08 to 0.58)	93 (2 observational studies)	⨁⨁⨁◯ MODERATE^c^	
Mortality in ICU-Hematological malignancy and BMT subgroup	543 per 1000	443 per 1000 (348 to 543)	OR 0.67 (0.45 to 1.00)	1816 (6 observational studies)	⨁◯◯◯ VERY LOW^d^	
Mortality in ICU Hematological malignancy and solid tumors subgroup	613 per 1000	222 per 1000 (137 to 337)	OR 0.18 (0.10 to 0.32)	239 (1 observational study)	⨁⨁◯◯ LOW	
30-day mortality	749 per 1000	503 per 1000 (396 to 616)	OR 0.34 (0.22 to 0.54)	335 (2 observational studies)	⨁⨁⨁◯ MODERATE^c^	

GRADE Working Group grades of evidence

High quality: We are very confident that the true effect lies close to that of the estimate of the effect

Moderate quality: We are moderately confident in the effect estimate: The true effect is likely to be close to the estimate of the effect, but there is a possibility that it is substantially different

Low quality: Our confidence in the effect estimate is limited: The true effect may be substantially different from the estimate of the effect

Very low quality: We have very little confidence in the effect estimate: The true effect is likely to be substantially different from the estimate of effect

*CI* confidence interval; *OR* odds ratio; *MD* mean difference

^a^The risk in the intervention group (and its 95 % confidence interval) is based on the assumed risk in the comparison group and the relative effect of the intervention (and its 95 % CI)

^b^Substantial heterogeneity may be due to methodological variations among studies and clinical variations among participants

^c^Upgraded due to large sample size and/or large outcome events

^d^95 % confidence interval up to 1

### Primary outcomes

**Fig. 2:**
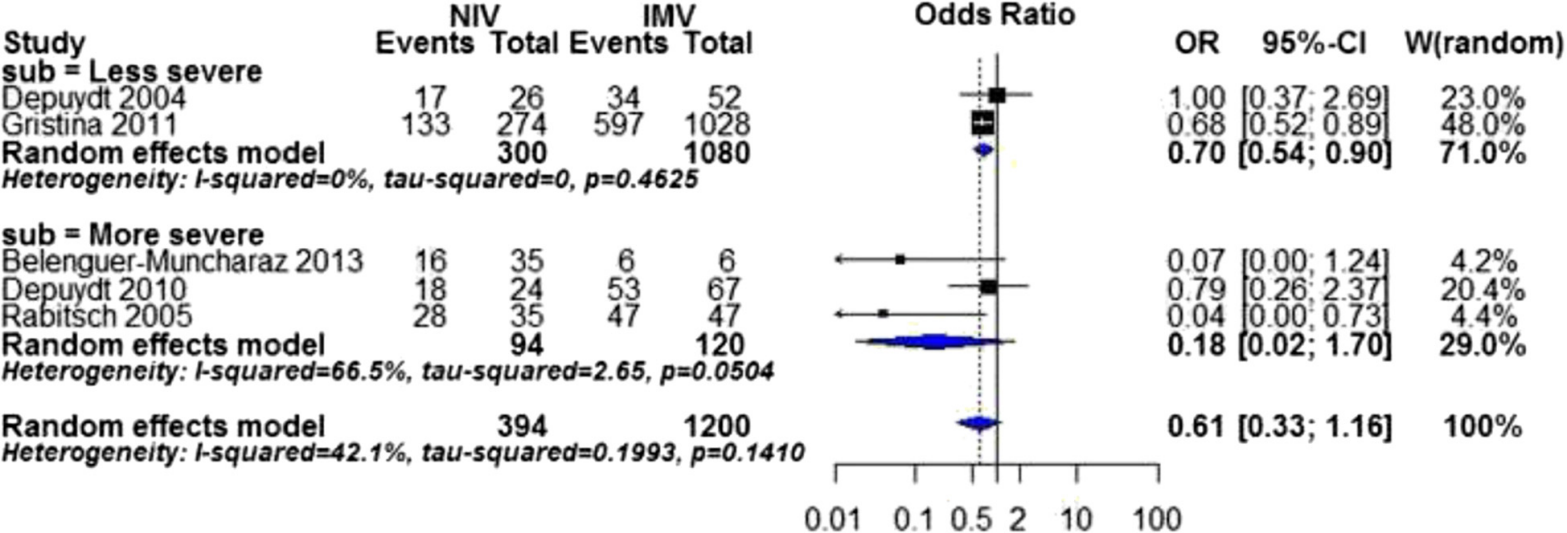
Mortality in hospital by disease severity. CI confidence interval, I2 percentage of total variation across studies from between-study heterogeneity rather than chance. *Vertical solid line* null effect, *Vertical dotted line* overall effect

We found a significantly lower mortality in hospital in NIV compared to IMV (OR 0.43, 95 % CI 0.23 to 0.80, *P* value = 0.007), but the heterogeneity was substantial (I^2^ statistic = 62 %). Subgroup analysis of less severe group showed similar result (OR 0.70, 95%CI 0.54 to 0.90, *P* value = 0.007; I^2^statistic = 0 %, Fig. 2). One study (Depuydt 2010) [34], with overlapped mean of SAPS II (52–65), was allocated in the more severe subgroup due to a substantially lower mean value of PaO2/FiO2 and relatively higher SOFA score than other studies. In AIDS subgroup, only one study was included, which showed NIV significantly reduced the mortality in hospital (OR 0.17, 95 % CI 0.03 to 0.81, *P* value = 0.03). In addition, multivariate logistic regression analysis reported in one study (Gristina 2011) [17], after adjustments for all available demographic characteristics and clinical variables, an initial NIV was associated with a significantly lower mortality in hospital (OR 0.73, 95 % CI 0.53 to 1.00, *P* value = 0.05).

**Fig. 3:**
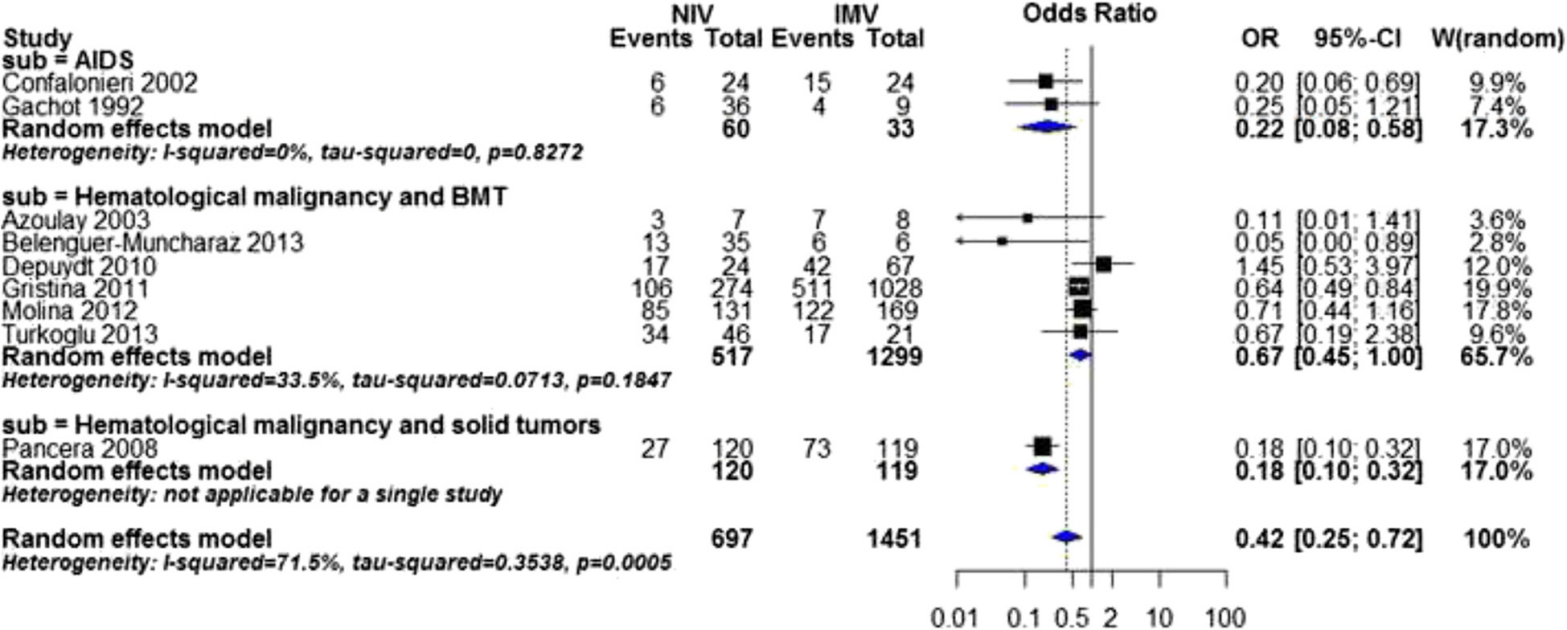
Mortality in ICU by cause of immunodeficiency

As for mortality in ICU, meta-analysis was not possible in both overall and subgroup analysis of disease severity due to substantial heterogeneity (overall analysis: I^2^ statistic = 72 %). Subgroup analysis of causes of immunodeficiency showed that NIV was associated with a significant reduction of mortality in ICU in all subgroups, AIDS subgroup (OR 0.20, 95 % CI 0.07 to 0.54, *P* value = 0.001, I^2^ statistic = 0 %, Fig. 3), hematological malignancy and BMT subgroup (OR 0.67, 95 % CI 0.45 to 1.00, *P* value = 0.05, I^2^ statistic = 34 %), and hematological malignancy and solid tumors subgroup (OR 0.18, 95 % CI 0.10 to 0.32, *P* value < 0.00001).

**Fig. 4:**
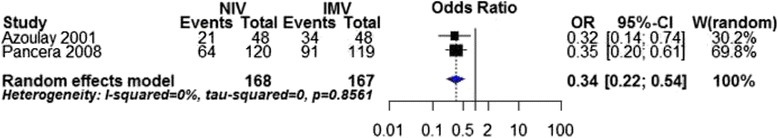
30-day mortality

The meta-analysis of two studies showed a statistically significant reduction of 30-day mortality between NIV versus IMV (OR 0.34, 95 % CI 0.22 to 0.54, *P* value < 0.0001, I^2^ statistic = 0 %, Fig. 4). Subgroup analysis of disease severity was not possible due to lacking of SAPS II data. Both studies were in the haematological malignancy and solid tumors subgroup, thus, significantly favour NIV. One study (Azoulay 2001) [28] reported a multivariate logistic regression analysis, showing that the probability of 30-day mortality was significantly decreased in NIV compared with IMV, with an odds ratio of 0.31 (95 % CI 0.12 to 0.82).

### Secondary outcomes

**Fig. 5:**
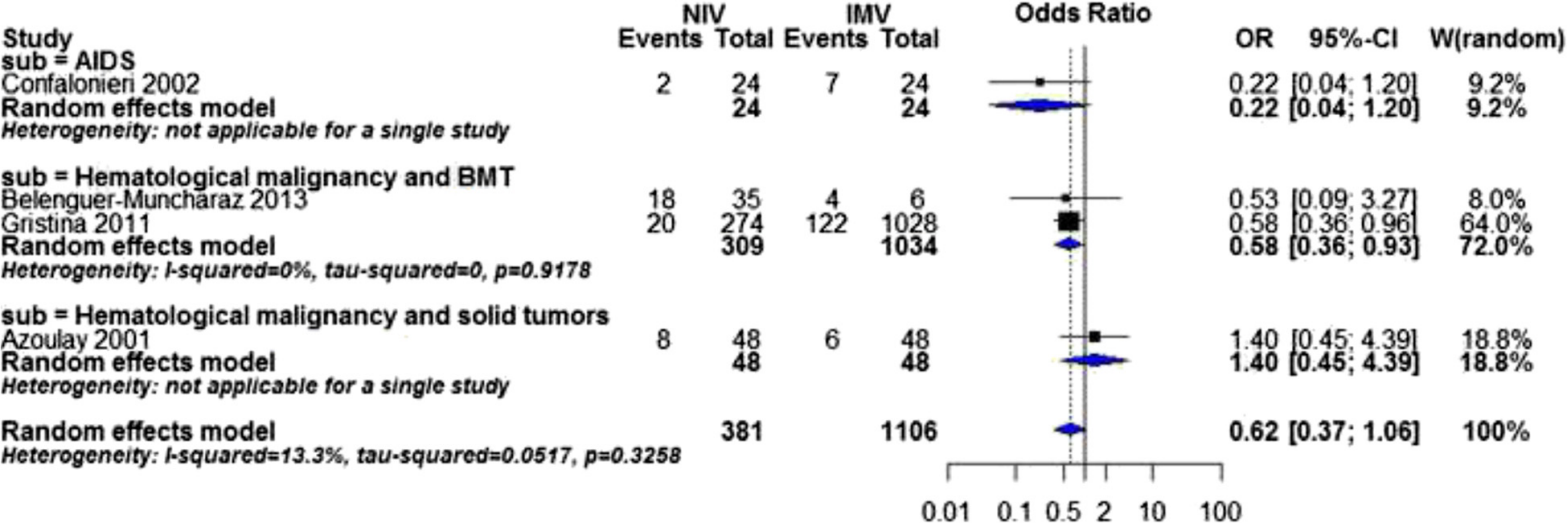
Nosocomial infections by cause of immunodeficiency

Four studies revealed no significant difference in the rate of nosocomial infections in patients on NIV in comparison with IMV. Similar results were shown in subgroup analysis of disease severity. Significant difference was found only in the hematological malignancy and BMT subgroup favoring NIV (OR 0.58, 95 % CI 0.36 to 0.93, *P* value = 0.03, I^2^ statistic = 0 %, Fig. 5).

Duration of mechanical ventilation was reported in three studies, but data couldn’t be pooled in the meta-analysis due to substantial heterogeneity (overall I^2^ statistic = 98 %). One study (Confalonieri 2002) [20] reported a statistically significant shortening of mechanical ventilation favoring NIV versus IMV (MD ± SD: 6 ± 2 versus 7 ± 1 days, *P* value = 0.034). Another (Gristina 2011) [17] reported that the mean duration of mechanical ventilation was 4 days (SD 4 days) in NIV group, compared to 11 days (SD 4 days) in IMV group (*P* value < 0.0001). The rest (B-M 2013) [36] revealed no difference among the two groups (*P* value = 0.08).

**Fig. 6:**
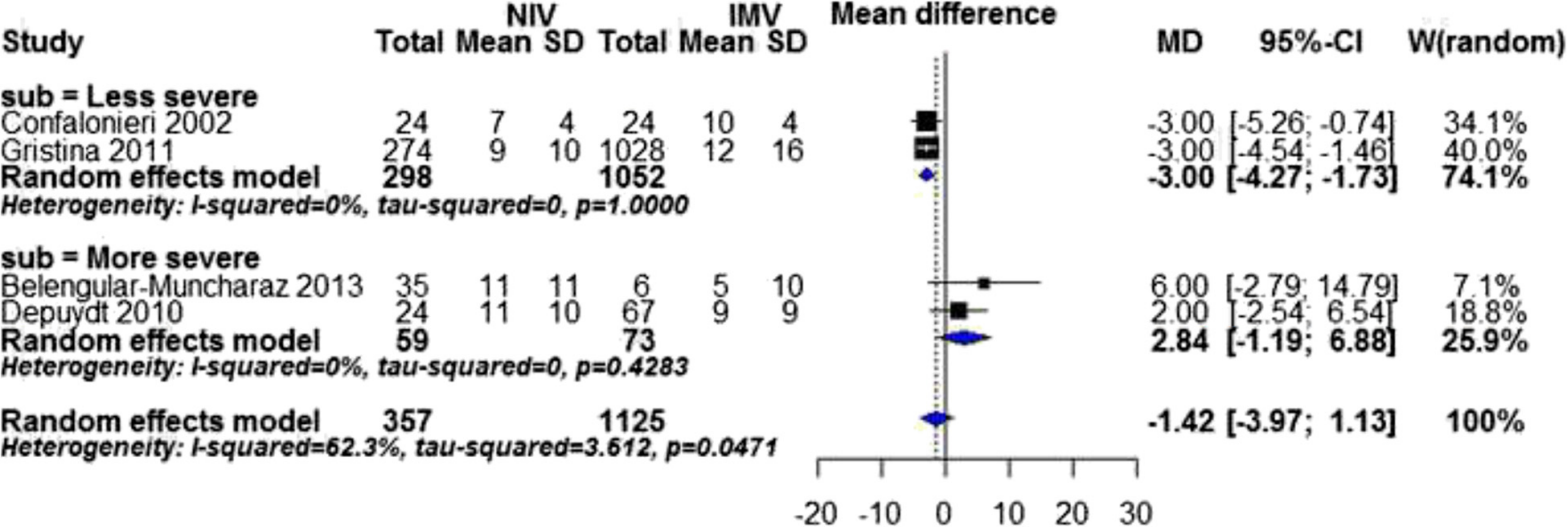
Duration of ICU stay by disease severity. SD standard deviation

Duration of ICU stay was reported in four studies. Overall and subgroup analyses of cause of immunodeficiency were not possible due to substantial heterogeneity (overall: I^2^ statistic = 62 %). In less severe subgroup, we found a statistically significant shortening of ICU stay in favor of NIV (MD −3.00 days; 95 % CI −4.27, −1.73 days, *P* value < 0.00001, I^2^ statistic = 0 %, Fig. 6).

**Fig. 7:**
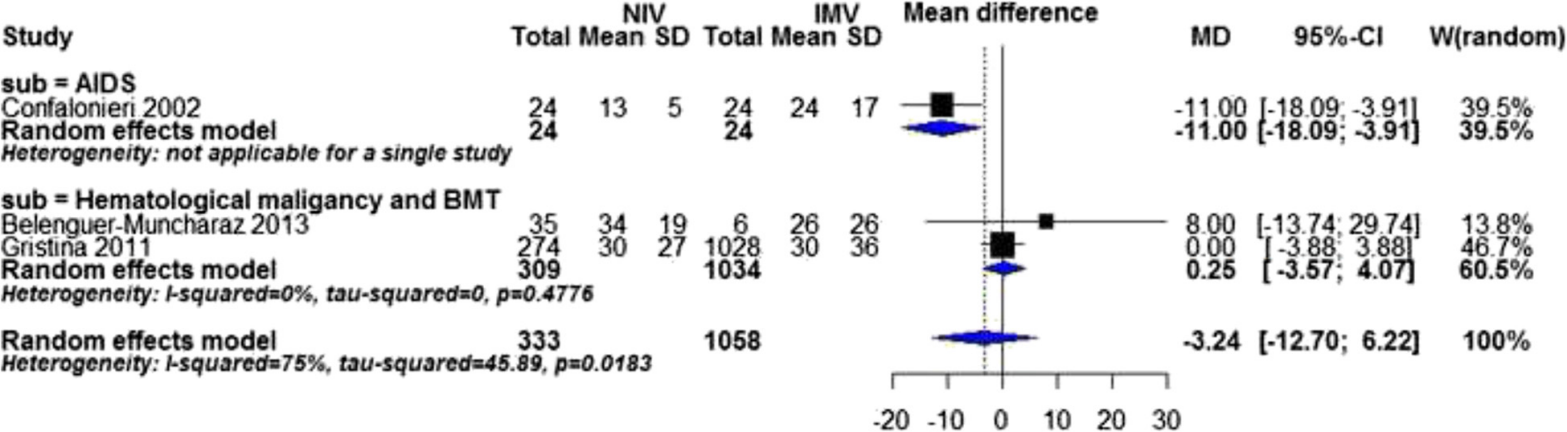
Duration of hospitalization by cause of immunodeficiency

Duration of hospitalization could only be pooled in the subgroup analysis of cause of immunodeficiency. One study (Confalonieri 2002) [20] in AIDS subgroup reported a statistically significant shortening of mechanical ventilation favoring NIV versus IMV (MD ± SD: 13 ± 5 versus 24 ± 17 days, *P* value = 0.004, Fig. 7).

## Discussion

To our knowledge this is the first systematic review aiming at compilation of the clinical evidence of the effect of NIV compared to IMV on ARF in immunocompromised patients. The overall evidence we obtained supports NIV over IMV in treating ARF in certain group of immunocompromised patients. Compared to IMV, NIV was shown to significantly reduce mortality in overall analysis, and mortality and duration of hospitalization/ICU stay in less severe (mainly reflected by SAPS II < 60), AIDS, hematological malignancy subgroups.

Compared with IMV, NIV is more frequently associated with minor complications because NIV avoids endotracheal tube or tracheostomy hence leaves the upper airway intact, and preserves airway defense mechanisms. Various complications directly related to the process of intubation and IMV such as nosocomial pneumonia, aspiration of gastric contents, ventilator-associated events, trauma of the teeth, hypopharynx, esophagus, larynx, and trachea could thus be avoided by application of NIV [19, 51, 52]. These complications are far more common in patients who are immunocompromised or critically ill. In addition, NIV causes less mask-related discomfort, unrecognized patient-ventilator asynchrony due to leaks, and milder gastric insufflations. At the meantime, NIV achieves the same physiological benefits of reduced work of breathing and improved gas exchange for certain groups of patients [53].

Although NIV was recommended as the first-line strategy for immunocompromised patients with ARF by guidelines in several countries [11, 54], our study along with several previous studies showed that NIV might not be the appropriate choice for all immunocompromised patients [15–17]. NIV has been stated by German and Canadian guidelines as the first-line treatment, with different recommendation levels, for immunocompromised patients with ARF [11, 54], though they were based on the same two RCTs [41, 55]. However, the control against NIV in both RCTs was standard oxygen therapy instead of IMV. In addition, participants in both RCTs were less severe than those in our review. The mean SAPS II in Hilbert 2001 [41] was no more than 45, and mean SAPS in Antonelli 2000 [55] was 13. Our result, along with several other reviews raised new puzzles and debates for NIV as the first-line approach for immunocompromised patients with ARF [16, 56]. We make the speculation that spectrum of choice for oxygen supplement strategy varies among standard oxygen therapy, NIV or IMV depending on different disease severity, and NIV or standard oxygen therapy should be applied in less severe patients. For the more severe patients, however, the choice might be limited within NIV or IMV. Our review showed that even in relatively more severe patients (45 < SAPS II < 60) than those in the two RCTs, NIV still showed significant advantages against IMV.

As is shown in our analysis, effects of NIV on patients vary with different levels of disease severity, different causes of immunocompromised status and types of ARF. Strict patient selection is of critical importance for the effect of NIV and other oxygen therapies. As for disease severity, our analysis showed NIV had clear advantage in less severe patients (mainly reflected by SAPS II < 60). For cause of immunodeficiency, NIV showed great advantage over IMV among AIDS patients in reducing mortality in both hospital and ICU, duration of both hospital and ICU stay, and also duration of mechanical ventilation. NIV was also related to better prognosis in hematological malignancies and BMT patients. Data available, though weak, also favored NIV in patients with solid tumors. One systematic review by Laura et al. [57] included thirteen studies to examine the effect of initial NIV versus IMV in hematological patients with ARF. Eight studies of which were also included in our review. NIV is associated with a lower risk of death in hematological patients (RR 0.74, 95 % CI 0.65 to 0.84, *p* < 0.0001), which was similar with our results. Unfortunately, there was little evidence showing the effects of NIV on different types of ARF due to lacking of data. Two studies [17, 38] showed poor prognosis of NIV in ALI/ARDS. NIV was strongly recommended as the first-line approach for acute exacerbation of chronic obstructive pulmonary disease (AECOPD), facilitation of weaning/extubation in patients with COPD and cardiogenic pulmonary edema in immunocompetent patients [11, 54]. The criteria between immunocompetent and immunocompromised patients are not identical, but similar. We expect similar efficacy of NIV in immunocompromised patients with these types of ARF. In addition, another review [58] emphasized the importance of patient selection and suggested both absolute and relative situations in which NIV is contraindicated. However, additional studies, especially randomized controlled trials, are needed to explore the possible benefits and/or risks of NIV in these groups of patients.

Considering that our analysis showed NIV reduced mortality in less severe patients, early recognition and prediction of NIV failure and timely initiation of IMV are of great importance for patients’ overall survival, especially for more severe patients. Laura et al. [57] showed that failure of NIV might worsen the prognosis, mainly in less severe patients. Predictors of failure of NIV in immunocompromised patients were summarised in one review [16], including higher illness severity at baseline reflected by SAPS II, higher respiratory rate under NIV, later initiation of NIV after ICU admission, need for vasopressors, renal replacement therapy, and presence of ALI/ARDS. Criteria used for NIV discontinuation and endotracheal intubation in immunocompromised patients were also suggested, including persistent dyspnea, severe hemodynamic or electrocardiographic instability, etc. The cut-off point for switching NIV to IMV in immunocompromised patients is still a puzzling issue which calls for more high-qualified studies [59].

The presence of patients who initially received NIV switched to IMV due to NIV failure did not substantially change our overall results and final conclusion. Almost all included studies (12 out of 13, excluding one study where the rate was not specified) reported such switching to IMV after initial NIV treatment. The mean intubation rate after initial NIV treatment was 49.5 %, greater than or equal to 50 % in seven studies and lower than 50 % in five studies (Table 1). We did a subgroup meta-analysis based on intubation rate in NIV group (Additional file 1), which showed that when intubation rate was lower than 50 %, NIV was more favorable in terms of mortality in ICU, 30-day mortality, duration of ICU stay as well as nosocomial infections. No significant difference was found between the NIV and IMV groups in terms of mortality in hospital, mortality in ICU and duration of ICU stay when intubation rate was greater than or equal to 50 %. It is thus reasonable to deduct that the final analysis results would be in even more favour of NIV if the overall intubation rate of all the studies included are lower than 50 %. In addition, a research where NIV patients are kept from switching to IMV once their conditions worsen would be unethical and in turn, impractical.

The major limitation of the present systematic review was that all included studies were exclusively observational. Given to the limitations of current data, RCTs of good methodological design are needed to address the effect of NIV versus IMV in treating acute respiratory failure in immunocompromised patients. Researchers should consider RCTs with a sample size large enough to demonstrate a meaningful result. Although it would be impossible to conduct double-blinded trials in the future due to the nature of intervention, it would be important to undertake blind assessment of the participants to ensure quality of the trials and minimize the risk of bias, with well classified causes of ARF and immunocompromised status and well stratified disease severity. Settings of intervention such as time between onset of acute respiratory failure and ICU admission, clear indications and contra-indications for NIV and or high flow oxygen therapy, type of interface and equipment use, strict NIV and high flow oxygen use protocol, early recognition of NIV or high flow oxygen failure, clear indications of intubation and invasive protective mechanical ventilation should also be well matched [56]. In place of RCTs, we suggest well-conducted observational studies, such as strictly matched prospective cohort studies.

## Conclusions

The overall evidence we obtained from 2552 immunocompromised patients with ARF shows NIV was associated with a significant lower mortality rates, especially in less severe patients and those who was immunosuppressed by AIDS, haematological malignancies and bone marrow transplant. For more severe patients, however, NIV didn’t show clear advantages over IMV. The advantages of NIV were also shown in reducing duration of hospitalization and ICU stay, as well as rates of nosocomial infection. Future studies need to be methodologically sound and include patients immunocompromised by other causes such as chemotherapy and receipt of glucocorticoids.

## Additional file

Additional file 1:
**Supplement 1.** Risk of bias graph: review authors’ judgements about each risk of bias item presented as percentages across all included studies. **Supplement 2.** Risk of bias summary: review authors’ judgements about each risk of bias item for each included study. **Supplement 3.** CENTRAL search strategy. **Supplement 4.** PubMed search strategy. **Supplement 5.** EMBASE search strategy. **Supplement 6.** CBM search strategy. **Supplement 7.** Assessment of risk of bias in cohort studies. **Supplement 8.** Assessment of risk of bias in case-control studies. **Supplement 9.** Newcastle-Ottawa Grading Results. **Supplement 10.** Subgroup meta-analysis based on intubation rate in NIV group. **Supplement 11.** Mortality in hospital by intubation rate. **Supplement 12.** Mortality in ICU by intubation rate. **Supplement 13.** 30-day mortality by intubation rate. **Supplement 14.** Duration of hospitalization by intubation rate. **Supplement 15.** Duration of ICU stay by intubation rate. **Supplement 16.** Nosocomial infection by intubation rate. **Supplement 17.** Duration of mechanical ventilaiton by intubation rate. (DOC 194 kb)

## Abbreviations

95 % CIs: 95 % confidence intervals
AECOPD: Acute exacerbation of chronic obstructive pulmonary disease
AIDS: Acquired immune deficiency syndrome
APACHE II: Acute physiology and chronic health evaluation II scores
ARDS/ALI: Adult respiratory distress syndrome/acute lung injury
ARF: Acute respiratory failure
Bi-PAP: Bi­level positive airway pressure
BMT: Bone marrow transplantation
CPAP: Continuous positive airway pressure
GRADE: The grading of recommendations assessment, development and evaluation
ICU: Intensive care unit
IMV: Invasive mechanical ventilation
MD: Mean difference
NIV: Noninvasive mechanical ventilation
OR: Odds ratio
PaO2/FiO2: Ratio of the partial pressure of arterial oxygen to the fraction of inspired oxygen
PEEP: Positive end expiratory pressure
PSV: Pressure support ventilation
RCTs: Randomized controlled trials
SAPS II: Simplified acute physiology scores II
SD: Standard deviation
SOFA: Sequential organ failure assessment
