# Supplementary material for: Noninvasive versus invasive mechanical ventilation for immunocompromised patients with acute respiratory failure: a systematic review and meta-analysis
Source: BMC Pulm Med. 2016 Aug 27;16(1):129. doi: 10.1186/s12890-016-0289-y (PMC5002326; doi:10.1186/s12890-016-0289-y)
Supplement: Additional file 1: — Supplement 1. Risk of bias graph: review authors’ judgements about each risk of bias item presented as percentages across all included studies. Supplement 2. Risk of bias summary: review authors’ judgements about each risk of bias item for each included study. Supplement 3. CENTRAL search strategy. Supplement 4. PubMed search strategy. Supplement 5. EMBASE search strategy. Supplement 6. CBM search strategy. Supplement 7. Assessment of risk of bias in cohort studies. Supplement 8. Assessment of risk of bias in case-control studies. Supplement 9. Newcastle-Ottawa Grading Results. Supplement 10. Subgroup meta-analysis based on intubation rate in NIV group. Supplement 11. Mortality in hospital by intubation rate. Supplement 12. Mortality in ICU by intubation rate. Supplement 13. 30-day mortality by intubation rate. Supplement 14. Duration of hospitalization by intubation rate. Supplement 15. Duration of ICU stay by intubation rate. Supplement 16. Nosocomial infection by intubation rate. Supplement 17. Duration of mechanical ventilaiton by intubation rate. (DOC 194 kb) [file 12890_2016_289_MOESM1_ESM.doc]

**Supplement 1.** Risk of bias graph: review authors' judgements about each risk of bias item presented as percentages across all included studies.


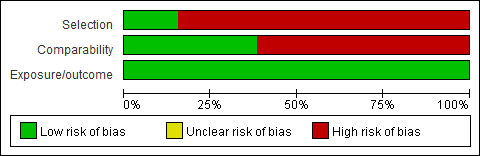


**Supplement 2.** Risk of bias summary: review authors' judgements about each risk of bias item for each included study.


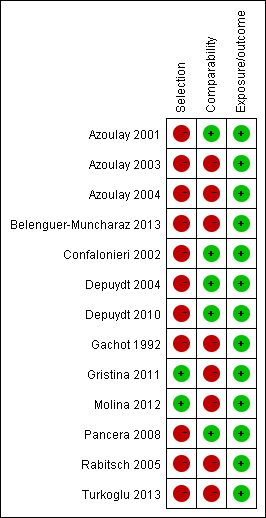


## Supplement 3. CENTRAL search strategy.

#1 MeSH descriptor: [Immunosuppression] explode all trees
#2 MeSH descriptor: [Immunocompromised Host] explode all trees
#3 MeSH descriptor: [Immunosuppressive Agents] explode all trees
#4 MeSH descriptor: [Immunologic Deficiency Syndromes] explode all trees
#5 MeSH descriptor: [Neutropenia] explode all trees
#6 MeSH descriptor: [Organ Transplantation] explode all trees
#7 MeSH descriptor: [Bone Marrow Transplantation] explode all trees
#8 MeSH descriptor: [Glucocorticoids] explode all trees
#9 MeSH descriptor: [Antineoplastic Agents] explode all trees
#10 MeSH descriptor: [HIV] explode all trees
#11 MeSH descriptor: [Acquired Immunodeficiency Syndrome] explode all trees
#12 MeSH descriptor: [Hematologic Neoplasms] explode all trees
#13 MeSH descriptor: [Cytotoxins] explode all trees
#14 (#1 or #2 or #3 or #4 or #5 or #6 or #7 or #8 or #9 or #10 or #11 or #12 or #13)
#15 immunosuppressed in All Text
#16 immunosuppressive in All Text
#17 immunodeficiency in All Text
#18 immunocompromised in All Text
#19 immunocompromise in All Text
#20 chemotherapy in All Text
#21 corticosteroid in All Text
#22 glucocorticoids therapy in All Text
#23 cytotoxic therapy in All Text
#24 Hematologic Malignancy in All Text
#25 AIDS in All Text
#26 immunosuppression in All Text
#27 Immunocompromised Host in All Text
#28 Immunosuppressive Agent* in All Text
#29 Immunologic Deficiency Syndromes in All Text
#30 neutropenia in All Text
#31 organ transplantation in All Text
#32 bone marrow transplantation in All Text
#33 glucocorticoid* in All Text
#34 Antineoplastic Agent* in All Text
#35 HIV in All Text
#36 Acquired Immunodeficiency Syndrome in All Text
#37 Hematologic Neoplasm* in All Text
#38 Cytotoxin* in All Text
#39 (#15 or #16 or #17 or #18 or #19 or #20 or #21 or #22 or #23 or #24 or #25 or #26 or #27 or #28 or #29 or #30 or #31 or #32 or #33 or #34 or #35 or #36 or #37 or #38)
#40 (#14 or #39)
#41 MeSH descriptor: [Acute Lung Injury] explode all trees
#42 MeSH descriptor: [Respiratory Distress Syndrome, Adult] explode all trees
#43 MeSH descriptor: [Pulmonary Disease, Chronic Obstructive] explode all trees
#44 MeSH descriptor: [Asthma] explode all trees
#45 MeSH descriptor: [Obesity Hypoventilation Syndrome] explode all trees
#46 MeSH descriptor: [Pulmonary Edema] explode all trees
#47 MeSH descriptor: [Pneumonia] explode all trees
#48 MeSH descriptor: [Lung Diseases, Interstitial] explode all trees
#49 acute respiratory failure in All Text
#50 chronic obstructive pulmonary disease in All Text
#51 cardiogenic pulmonary edema in All Text
#52 Diffuse Parenchymal Lung Disease in All Text
#53 interstitial lung disease* in All Text
#54 ARF in All Text
#55 ALI in All Text
#56 ARDS in All Text
#57 COPD in All Text
#58 OHS in All Text
#59 CPE in All Text
#60 DPLD in All Text
#61 acute lung injury in All Text
#62 Acute Respiratory Distress Syndrome in All Text
#63 asthma in All Text
#64 obesity hypoventilation syndrome in All Text
#65 pulmonary edema in All Text
#66 pneumonia in All Text
#67 (#41 or #42 or #43 or #44 or #45 or #46 or #47 or #48 or #49 or #50 or #51 or #52 or #53 or #54 or #55 or #56 or #57 or #58 or #59 or #60 or #61 or #62 or #63 or #64 or #65 or #66)
#68 MeSH descriptor: [Respiration, Artificial] explode all trees
#69 MeSH descriptor: [Intubation, Intratracheal] explode all trees
#70 mechanical ventilation in All Text
#71 artificial respiration in All Text
#72 artificial ventilation in All Text
#73 assisted ventilation in All Text
#74 Continuous positive airway pressure ventilation in All Text
#75 endotracheal ventilation in All Text
#76 endotracheal intubation in All Text
#77 pressure support ventilation in All Text
#78 positive end expiratory pressure in All Text
#79 bi-level positive airway pressure in All Text
#80 bilevel positive airway pressure in All Text
#81 invasive positive pressure ventilation in All Text
#82 non invasive ventilation in All Text
#83 non invasive positive pressure ventilation in All Text
#84 bipap in All Text
#85 nippv in All Text
#86 nppv in All Text
#87 niv in All Text
#88 niav in All Text
#89 aprv in All Text
#90 cpap in All Text
#91 ippb in All Text
#92 ippv in All Text
#93 peep in All Text
#94 (#68 or #69 or #70 or #71 or #72 or #73 or #74 or #75 or #76 or #77 or #78 or #79 or #80 or #81 or #82 or #83 or #84 or #85 or #86 or #87 or #88 or #89 or #90 or #91 or #92 or #93)
#95 (#40 and #67 and #94)

## Supplement 4. PubMed search strategy.

1. "immunosuppression"[MeSH]
2. "Immunocompromised Host"[MeSH]
3. "Immunologic Deficiency Syndromes"[MeSH]
4. "neutropenia"[MeSH]
5. "organ transplantation "[MeSH]
6. "bone marrow transplantation "[MeSH]
7. "glucocorticoids "[MeSH]
8. "Glucocorticoids "[Pharmacological Action]
9. "Antineoplastic Agents "[MeSH]
10. "HIV"[MeSH]
11. "Hematologic Neoplasms"[MeSH]
12. "Hematologic Neoplasms"[All Fields] "immunosuppression"[All Fields] OR"Immunocompromised Host"[All Fields] OR"Immunologic Deficiency Syndromes"[All Fields] OR"neutropenia"[All Fields] OR"organ transplantation"[All Fields] OR"bone marrow transplantation"[All Fields] OR"glucocorticoids "[All Fields] OR"Antineoplastic Agents"[All Fields] OR"HIV"[All Fields] OR"immunosuppressed"[All Fields] OR"immunosuppressive"[All Fields] OR"immunodeficiency"[All Fields] OR"immunocompromised "[All Fields] OR"immunocompromise"[Title/Abstract] OR"chemotherapy "[Title/Abstract] OR"corticosteroid"[Title/Abstract] OR"glucocorticoids therapy"[Title/Abstract] OR"cytotoxic therapy"[Title/Abstract] OR"acquired immunodeficiency syndrome "[All Fields] OR"AIDS"[Title/Abstract]
13. 1 or 2-12 1900079
14. "acute lung injury"[MeSH]
15. "Respiratory Distress Syndrome, Adult"[MeSH]
16. "Pulmonary Disease, Chronic Obstructive"[MeSH]
17. "asthma "[MeSH]
18. "obesity hypoventilation syndrome"[MeSH]
19. "pulmonary edema "[MeSH]
20. "pneumonia"[MeSH]
21. "Pneumonia, Ventilator-Associated"[MeSH]
22. "Lung Diseases, Interstitial"[MeSH]
23. "acute lung injury"[All Fields] OR"Respiratory Distress Syndrome, Adult"[All Fields] OR"Pulmonary Disease, Chronic Obstructive"[All Fields] OR"asthma"[All Fields] OR"obesity hypoventilation syndrome"[Title/Abstract] OR"pulmonary edema"[All Fields] OR"pneumonia"[All Fields] OR"Pneumonia, Ventilator-Associated"[All Fields] OR"Lung Diseases, Interstitial"[All Fields] OR"acute respiratory failure "[All Fields] OR"ARF"[Title/Abstract] OR"ALI"[Title/Abstract] OR"acute respiratory distress syndrome "[All Fields] OR"ARDS"[Title/Abstract] OR"chronic obstructive pulmonary disease"[All Fields] OR"COPD"[Title/Abstract] OR"OHS"[Title/Abstract] OR"cardiogenic pulmonary edema "[All Fields] OR"CPE"[Title/Abstract] OR"interstitial lung disease"[All Fields] OR"Diffuse Parenchymal Lung Disease"[All Fields] OR"DPLD"[Title/Abstract]
24. 14 or 15-23 424223
25. "Respiration, Artificial"[Mesh]
26. "noninvasive ventilation"[MeSH]
27. "Intubation, Intratracheal"[MeSH]
28. "High-Frequency Ventilation"[MeSH]
29. "Respiration, Artificial"[All Fields] OR"mechanical ventilation"[All Fields] OR"artificial respiration"[All Fields] OR"noninvasive ventilation"[All Fields] OR"Intubation, Intratracheal"[All Fields] OR"High-Frequency Ventilation"[All Fields] OR"Continuous positive airway pressure ventilation "[All Fields] OR"endotracheal ventilation "[All Fields] OR"endotracheal intubation "[All Fields] OR"artificial ventilation "[All Fields] OR"assisted ventilation "[Title/Abstract] OR"bipap "[Title/Abstract] OR"nippv "[Title/Abstract] OR"nppv "[Title/Abstract] OR"niv "[Title/Abstract] OR"niav "[Title/Abstract] OR"cpap "[Title/Abstract] OR"aprv "[Title/Abstract] OR"ippb "[Title/Abstract] OR"ippv "[Title/Abstract] OR"peep "[Title/Abstract] OR"pressure support ventilation "[Title/Abstract] OR"positive end expiratory pressure "[Title/Abstract] OR"bi-level positive airway pressure "[Title/Abstract] OR"bilevel positive airway pressure "[Title/Abstract] OR"invasive positive pressure ventilation"[All Fields] OR"non invasive ventilation "[All Fields] OR"non invasive positive pressure ventilation"[All Fields]
30. 25 or 26-29 113950
31 ("Animals"[Mesh]) NOT "Humans"[Mesh]
32 13 and 24 and 30 not 31

## Supplement 5. EMBASE search strategy.

1. 'immune deficiency'/exp
2. 'immunocompromised patient'/exp
3. 'immunosuppressive treatment'/exp
4. 'immunosuppressive agent'/exp
5. 'antineoplastic agent'/exp
6. 'neutropenia'/exp
7. 'organ transplantation'/exp
8. 'bone marrow transplantation'/exp
9. 'glucocorticoid'/exp
10. 'human immunodeficiency virus'/exp
11. 'human immunodeficiency virus infection'/exp (including acquired immune deficiency syndrome)
12. 'hematologic malignancy'/exp
13. 'chemotherapy'/exp
14. 'corticosteroid'/exp
15. 'immune deficiency' OR 'immunocompromised patient' OR 'immunosuppressive treatment' OR 'immunosuppressive agent' OR 'antineoplastic agent' OR 'neutropenia' OR 'organ transplantation' OR 'bone marrow transplantation' OR 'glucocorticoid' OR 'human immunodeficiency virus' OR 'human immunodeficiency virus infection' OR 'acquired immune deficiency syndrome' OR 'hematologic malignancy' OR 'chemotherapy' OR 'corticosteroid' OR 'immunosuppression' OR 'immunocompromised host' OR 'immunologic deficiency syndromes' OR 'glucocorticoids' OR 'antineoplastic agents' OR 'hiv' OR 'hematologic neoplasms' OR 'immunosuppressed' OR 'immunosuppressive' OR 'immunodeficiency' OR 'immunocompromised' OR 'immunocompromise' OR 'glucocorticoids therapy' OR 'cytotoxic therapy' OR 'acquired immunodeficiency syndrome' OR 'aids'
16. 1 or 2-15 3338045
17. 'respiratory distress syndrome'/exp (including acute lung injury & adult respiratory distress syndrome)
18. 'chronic obstructive lung disease'/exp
19. 'asthma'/exp
20. 'obesity hypoventilation syndrome'/exp
21. 'lung edema'/exp
22. 'pneumonia'/exp (including ventilator associated pneumonia)
23. 'interstitial lung disease'/exp
24. 'acute respiratory failure'/exp
25. 'adult respiratory distress syndrome' OR 'chronic obstructive lung disease' OR 'asthma' OR 'obesity hypoventilation syndrome' OR 'lung edema' OR 'pneumonia' OR 'ventilator associated pneumonia' OR 'interstitial lung disease' OR 'acute respiratory failure' OR 'acute lung injury' OR 'respiratory distress syndrome' OR 'chronic obstructive pulmonary disease' OR 'pulmonary edema' OR 'cardiogenic pulmonary edema' OR 'diffuse parenchymal lung disease' OR 'arf' OR 'ali' OR 'ards' OR 'copd' OR 'ohs' OR 'cpe' OR 'dpld'
26. 17 or 18-25 813284
27. 'artificial ventilation'/exp (including noninvasive ventilation)
28. 'respiratory tract intubation'/exp (including endotracheal intubation)
29. 'assisted ventilation'/exp
30. 'ventilator'/exp
31. 'artificial ventilation' OR 'respiratory tract intubation' OR 'assisted ventilation' OR 'ventilator' OR 'mechanical ventilation' OR 'artificial respiration' OR 'noninvasive ventilation' OR 'non invasive ventilation' OR 'intratracheal intubation' OR 'high-frequency ventilation' OR 'continuous positive airway pressure ventilation' OR 'endotracheal ventilation' OR 'endotracheal intubation' OR 'bipap' OR 'nippv' OR 'nppv' OR 'niv' OR 'niav' OR 'cpap' OR 'aprv' OR 'ippb' OR 'ippv' OR 'peep' OR 'pressure support ventilation' OR 'positive end expiratory pressure' OR 'bi-level positive airway pressure' OR 'bilevel positive airway pressure' OR 'invasive positive pressure ventilation' OR 'non invasive positive pressure ventilation'
32. 27 or 28-31 229096
33. 'nonhuman'/exp OR 'animal'/exp
34. 16 and 26 and 32 not 33

## Supplement 6. CBM search strategy.

61 ((#59) AND (#45)) AND ( 人类[特征词]) 563
60 (#59) AND (#45) 574 20:58:58
59 (#58) OR (#57) OR (#56) OR (#55) OR (#54) OR (#53) OR (#52) OR (#51) OR (#50) OR (#49) OR (#48) OR (#47) OR (#46) 74561
58 气管内插管 2774
57 气管插管 26497
56 人工辅助通气 32
55 人工呼吸 1219
54 有创机械通气 1700
53 无创机械通气 1462
52 有创通气 1156
51 无创通气 2968
50 机械通气 29142
49 "插管法, 气管内"[不加权:扩展] 21858
48 "连续气道正压通气"[不加权:扩展] 1587
47 "通气机, 机械"[不加权:扩展] 11667
46 "呼吸, 人工"[不加权:扩展] 33806
45 (#44) AND (#24) 10856
44 (#42) OR (#41) OR (#40) OR (#39) OR (#38) OR (#37) OR (#36) OR (#35) OR (#34) OR (#33) OR (#32) OR (#31) OR (#30) OR (#29) OR (#28) OR (#27) OR (#26) OR (#25) 243723
43 "成人呼吸窘迫综合征"[全字段:智能] 16788
42 成人呼吸窘迫综合征 16788
41 急性肺损伤 16788
40 间质性肺病 400
39 间质性肺炎 2158
38 弥漫性肺间质病 16
37 心源性肺水肿 731
36 慢性阻塞性肺病 7954
35 呼吸衰竭 42797
34 急性呼吸衰竭 2279
33 "肺疾病, 间质性"[不加权:扩展] 25092
32 "肺炎, 呼吸机相关性"[不加权:扩展] 3394
31 "肺炎"[不加权:扩展] 78503
30 "肺水肿"[不加权:扩展] 7398
29 "肥胖低通气综合征"[不加权:扩展] 42
28 "哮喘"[不加权:扩展] 55060
27 "肺疾病, 慢性阻塞性"[不加权:扩展] 35057
26 "呼吸窘迫综合征, 成人"[不加权:扩展] 13090
25 "呼吸功能不全"[不加权:扩展] 31616
24 (#23) OR (#22) OR (#21) OR (#20) OR (#19) OR (#18) OR (#17) OR (#16) OR (#15) OR (#14) OR (#13) OR (#12) OR (#11) OR (#10) OR (#9) OR (#8) OR (#7) OR (#6) OR (#5) OR (#4) OR (#3) OR (#2) OR (#1) 338425
23 艾滋病 46114
22 AIDS 46114
21 血液系统恶性肿瘤 737
20 皮质醇类 19
19 免疫减低 2
18 免疫减退 4
17 免疫缺陷 39950
16 免疫抑制 25592
15 化疗 161484
14 "细胞毒素类"[不加权:扩展] 344
13 "血液肿瘤"[不加权:扩展] 3233
12 "HIV"[不加权:扩展] 17134
11 "HIV感染"[不加权:扩展] 40018
10 "抗肿瘤药"[不加权:扩展] 28171
9 "糖皮质激素类"[不加权:扩展] 13522
8 "骨髓移植"[不加权:扩展] 6785
7 "器官移植"[不加权:扩展] 56607
6 "中性粒细胞减少"[不加权:扩展] 595
5 "免疫抑制剂"[不加权:扩展] 8458
4 "免疫抑制法"[不加权:扩展] 6121
3 "获得性免疫缺陷综合征"[不加权:扩展] 32212
2 "免疫缺陷综合征"[不加权:扩展] 42349
1 "免疫减弱宿主"[不加权:扩展] 41

## Supplement 7. Assessment of risk of bias in cohort studies

**Adapted Newcastle-Ottawa Scale for cohort studies:**
We will classify studies as low risk of bias (up to one inadequate item in the NOS), medium risk of bias (up to three inadequate items) and high risk of bias (more than three inadequate or no description of methods). The following items will be assessed:

**Selection**

1) Representativeness of the Exposed Cohort ( patients diagnosed with acute respiratory failure, whose immune status is well characterized)

a) truly representative of the exposed cohort *
b) somewhat representative of the exposed cohort *
c) selected group of exposed immunocompromised patients with ARF
d) no description of the derivation of the cohort

2) Selection of the Non-Exposed Cohort

a) drawn from the same community as the exposed cohort *
b) drawn from a different source
c) no description of the derivation of the non exposed cohort

3) Ascertainment of Exposure

a) secure record *
b) structured interview *
c) written self report
d) no description

4) Demonstration That Outcome of Interest Was Not Present at Start of Study

a) yes *
b) no

**Comparability**

1) Comparability of Cohorts on the Basis of the Design or Analysis

A maximum of 2 stars can be allotted in this category

a) study controls for demographic characteristics (e.g. age, sex) *
b) study controls for functional status (e.g. SAPS II, PaO2:FiO2) *
c) statements of no differences between groups or that differences were not statistically significant are not sufficient
d) no description

**Outcome**

1) Assessment of Outcome

a) independent or blind assessment *
b) record linkage *
c) self-report
d) no description

2) Was Follow-Up Long Enough for Outcomes to Occur

a) yes (select an adequate follow up period for outcome of interest) *
b) no

3) Adequacy of Follow Up of Cohorts

a) complete follow up *
b) subjects lost to follow up unlikely to introduce bias(i.e. follow up rate > 80% or description provided of those lost) *
c) follow up rate < 80% and no description of those lost
d) no description

## Supplement 8. Assessment of risk of bias in case-control studies.

**Adapted Newcastle-Ottawa Scale for case-control studies:**
We will classify studies as low risk of bias (up to one inadequate item in the NOS), medium risk of bias (up to three inadequate items) and high risk of bias (more than three inadequate or no description of methods). The following items will be assessed:

**Selection**

1) Is the Case Definition Adequate?

a) Requires some independent validation (e.g. >1 person/record/time/process to extract information, or reference to primary record source such as medical/hospital records) *
b) Record linkage or self-report with no reference to primary record
c) No description

2) Representativeness of the Cases

a) truly representative of the exposed population *
b) not satisfying requirements in part (a), or not stated

3) Selection of Controls

a) drawn from the same community as the exposed group *
b) drawn from a different source
c) no description

4) Definition of Controls

a) If cases are first occurrence of outcome, then it must explicitly state that controls have no history of this outcome. If cases have new (not necessarily first) occurrence of outcome, then controls with previous occurrences of outcome of interest should not be excluded.*
b) No mention of history of outcome

**Comparability**

1) Comparability of Cases and Controls on the Basis of the Design or Analysis

A maximum of 2 stars can be allotted in this category

a) study controls for demographic characteristics (e.g. age, sex) *
b) study controls for functional status (e.g. SAPS II, PaO2:FiO2) *
c) statements of no differences between groups or that differences were not statistically significant are not sufficient
d) no description

**Exposure**

1) Ascertainment of Exposure

a) independent or blind assessment *
b) record linkage *
c) self-report
d) no description

2) Non-Response Rate

a) complete follow up *
b) subjects lost to follow up unlikely to introduce bias(i.e. follow up rate > 80% or description provided of those lost) *
c) follow up rate < 80% and no description of those lost
d) no description

## Supplement 9. Newcastle-Ottawa Grading Results.

| Studies | Study type | Selection score | Comparability score | Exposure/outcome score | Total stars score | Risk level |
| --- | --- | --- | --- | --- | --- | --- |
| [Azoulay 2001](../Azoulay%202001) | Cohort | 2 | 2 | 3 | 7 out of 9 | Medium |
| [Confalonieri 2002](../Confalonieri%202002) | Cohort | 2 | 2 | 3 | 7 out of 9 | Medium |
| [Depuydt 2004](../Depuydt%202004) | Cohort | 2 | 3 | 3 | 8 out of 9 | Low |
| [Depuydt 2010](../Depuydt%202010) | Cohort | 2 | 2 | 3 | 7 out of 9 | Medium |
| [Gachot 1992](../Gachot%201992) | Case-control | 2 | 0 | 1 | 3 out of 8 | High |
| [Gristina 2011](../Gristina%202011) | Case-control | 3 | 0 | 2 | 5 out of 8 | High |
| [Pancera 2008](../Pancera%202008) | Case-control | 2 | 1 | 2 | 5 out of 8 | High |
| [Rabitsch 2005](../Rabitsch%202005) | Case-control | 1 | 0 | 2 | 3 out of 8 | High |
| [Azoulay 2003](../Azoulay%202003) | Case-control | 2 | 0 | 2 | 4 out of 8 | High |
| [Azoulay 2004](../Azoulay%202004) | Case-control | 2 | 0 | 2 | 4 out of 8 | High |
| [B-M 2013](../Belenguer-Muncharaz%202013) | Case-control | 2 | 0 | 2 | 4 out of 8 | High |
| [Molina 2012](../Molina%202012) | Case-control | 4 | 0 | 2 | 6 out of 8 | Medium |
| [Turkoglu 2013](../Turkoglu%202013) | Case-control | 2 | 0 | 2 | 4 out of 8 | High |

## Supplement 10. Subgroup meta-analysis based on intubation rate in NIV group.

| *Outcomes* | *Subgroups(intubation rate in NIV group)* | *Number of studies* | *OR (95%CIs)* | *P value* | *Heterogeneity(I^2^)* |
| --- | --- | --- | --- | --- | --- |
| Mortality(in hospital) | <50% | 3 | 0.31(0.08,1.08) | 0.09 | Substantial(62%) |
|  | ≥50% | 4 | 0.44(0.17,1.17) | 0.10 | Substantial(65%) |
| Mortality(in ICU) | <50% | 5 | 0.25(0.11,0.61) | 0.002 | Substantial(81%) |
|  | ≥50% | 4 | 0.76(0.44,1.34) | 0.35 | Little(23%) |
| 30-day mortality | <50% | 1 | 0.35(0.20,0.61) | 0.0002 | Not appliable |
|  | ≥50% | 0 |  |  |  |
| Nosocomial infections | <50% | 3 | 0.54(0.34,0.86) | 0.009 | Little(0%) |
|  | ≥50% | 0 |  |  |  |
| Duration of ICU stay | <50% | 3 | -2.48(-4.68,-0.27) | 0.03 | Medium(49%) |
|  | ≥50% | 1 | 2.00(-2.54,6.54) | 0.39 | Not appliable |
| Duration of hospitalization | <50% | 3 | -3.24(-3.88,3.88) | 0.50 | Substantial(75%) |
|  | ≥50% | 0 |  |  |  |
| Duration of mechanical ventilation | <50% | 3 | -1.66(-6.87,3.55) | 0.53 | Substantial(98%) |
|  | ≥50% | 0 |  |  |  |

OR: Odds ration; 95% CIs: 95% confidence intervals


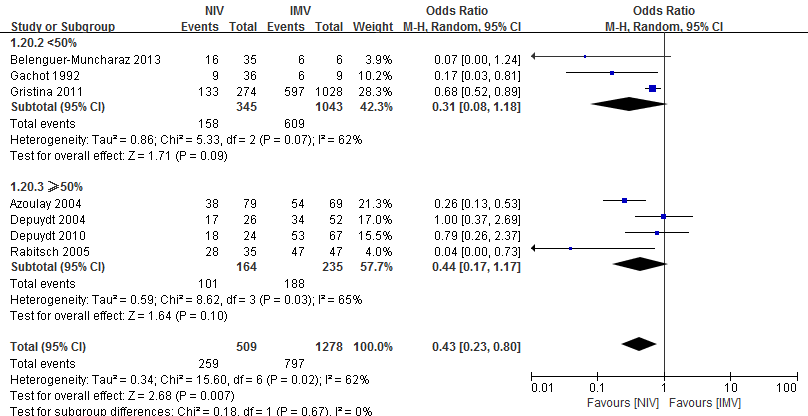


**Supplement 11.** Mortality in hospital by intubation rate.


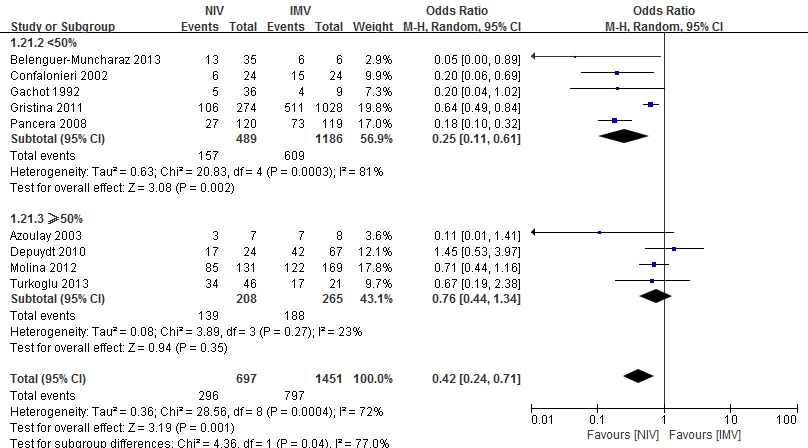


**Supplement 12.** Mortality in ICU by intubation rate.


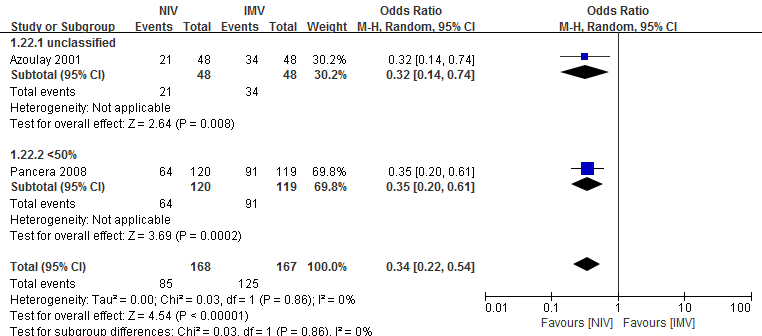


**Supplement 13.** 30-day mortality by intubation rate.


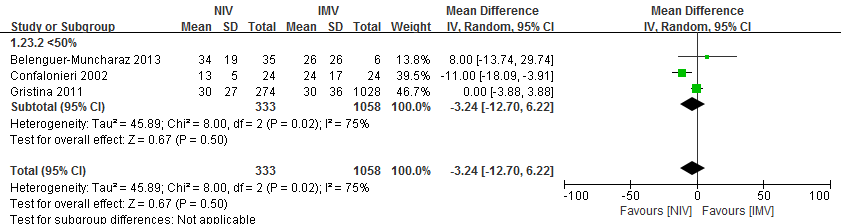


**Supplement 14.** Duration of hospitalization by intubation rate.


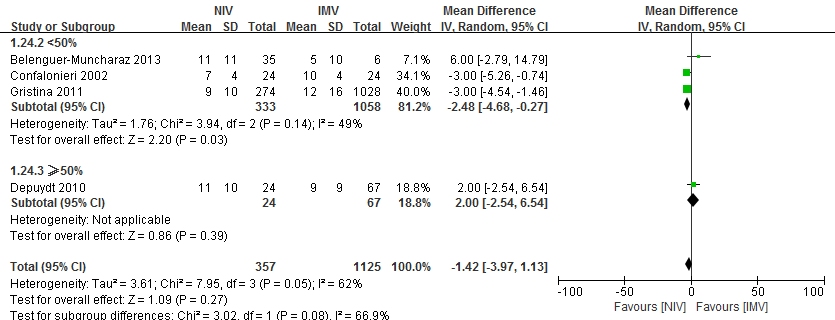


**Supplement 15.** Duration of ICU stay by intubation rate.


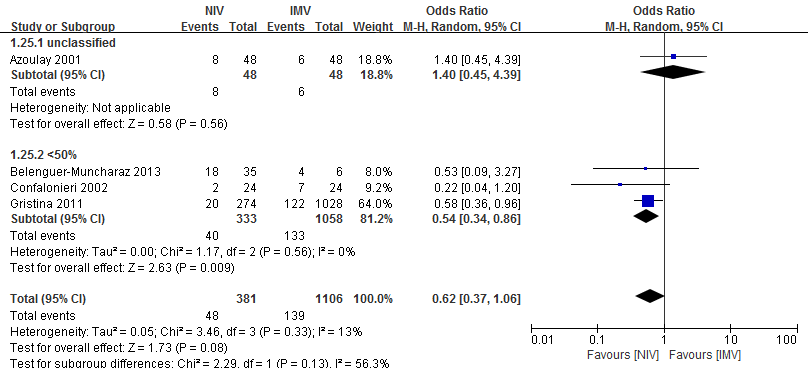


**Supplement 16.** Nosocomial infection by intubation rate.


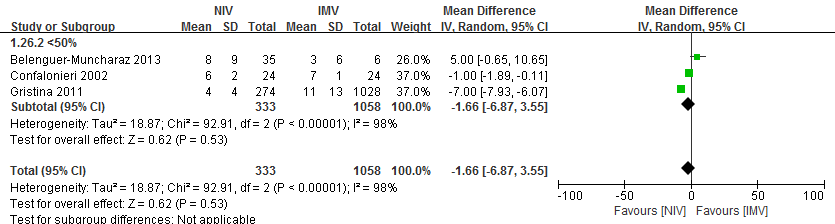


**Supplement 17.** Duration of mechanical ventilaiton by intubation rate.
